# Design of chemical space networks incorporating compound distance relationships

**DOI:** 10.12688/f1000research.10021.2

**Published:** 2016-11-30

**Authors:** Antonio de la Vega de León, Jürgen Bajorath

**Affiliations:** 1 Department of Life Science Informatics, Rheinische Friedrich-Wilhelms-Universität, Bonn, Germany

**Keywords:** Chemical space, bioactive compounds, coordinate-free space representations, chemical similarity, similarity-based compound networks, force-directed network layouts

## Abstract

Networks, in which nodes represent compounds and edges pairwise similarity relationships, are used as coordinate-free representations of chemical space. So-called chemical space networks (CSNs) provide intuitive access to structural relationships within compound data sets and can be annotated with activity information. However, in such similarity-based networks, distances between compounds are typically determined for layout purposes and clarity and have no chemical meaning. By contrast, inter-compound distances as a measure of dissimilarity can be directly obtained from coordinate-based representations of chemical space. Herein, we introduce a CSN variant that incorporates compound distance relationships and thus further increases the information content of compound networks. The design was facilitated by adapting the Kamada-Kawai algorithm. Kamada-Kawai networks are the first CSNs that are based on numerical similarity measures, but do not depend on chosen similarity threshold values.

## Introduction

In chemoinformatics, molecular network representations have thus far mostly been applied to study similarity relationships between compounds and visualize structure-activity relationships (SARs) ^1– 3^. In such networks, molecules are represented as nodes and edges indicate pairwise similarity relationships. Potency information can be added, for example, through node coloring, which provides a basis for SAR visualization ^2^. A prototypic network representation specifically designed for SAR analysis was the ‘network-like similarity graph’ (NSG) ^3^, a precursor of more generally defined ‘chemical space networks’ (CSNs) ^4^, which are characterized using statistical concepts from the interdisciplinary field of network science ^5^. As SAR-oriented network representations, NSGs provide immediate visual access to local communities (subsets) of active compounds with interesting SAR characteristics.

A major distinguishing feature of different CSNs is the way in which molecular similarity relationships are established ^5^. The use of alternative similarity measures often changes local and global network properties of CSNs ^5^. When numerical similarity measures are used, pairwise compound comparisons yield a similarity matrix that contains similarity values for all compound pairs in a data set. The application of a similarity threshold value then transforms the similarity matrix into an adjacency matrix, which serves as input for layout algorithms to generate a graphical representation ^6^. In fact, network appearance is often strongly influenced by chosen layout algorithms.

Conventional chemical space representations used in chemoinformatics are mostly generated on the basis of vectors of numerical descriptors. The resulting coordinate-based space representations are multi- or high-dimensional, with each chosen descriptor adding another dimension to the space. In such coordinate-based spaces, compound positions are unambiguously defined and so are distances between compounds that are quantified as a measure of dissimilarity, i.e. the larger the distance is, the more dissimilar the compounds are. By contrast, CSNs have become a paradigm of coordinate-free chemical space representations, which are entirely determined by pairwise similarity relationships ^4, 5^. If substructure-based similarity measures are employed, binary relationships are obtained (i.e. two compounds are either ‘similar’ or not); if similarity threshold values are applied to numerical measures, pairs of compounds reaching the threshold are classified as similar (and appear in the adjacency matrix). Hence, distance relationships between compounds are typically not considered in coordinate-free chemical space representations.

In this work, we introduce a novel layout for CSNs that does not depend on chosen threshold values, but takes distances derived from pairwise similarity values into account. Thus, in contrast to currently available CSNs, distances between compounds and communities in the resulting networks become chemically relevant (at least with respect to chosen descriptors), which further increases the information content of these representations.

## Methods

### Data sets

For network design, one large and three small compound sets (active against human targets with defined equilibrium constants) were taken from ChEMBL (version 21) ( https://www.ebi.ac.uk/chembl/) ^7^, as reported in Table 1. We note that there was no specific reason to focus on these sets; many others could have been selected instead.

**Table 1.** Compound sets.

ID	Target set	# CPDs
11638	MAP kinase ERK2 inhibitors	90
222	Glutamate [NMDA] receptor subunit ε 2 ligands	59
100476	Apoptosis regulator Bcl-W inhibitors	48
51	Serotonin 1a (5-HT1a) receptor ligands	1680

‘ID’ is the ChEMBL target identifier and ‘# CPDs’ means number of compounds.

### Molecular representation and similarity metric

Compounds were represented using the MACCS fingerprint ^8^ (consisting of 166 structural keys or patterns), which were generated using an in-house Python implementation. Pairwise similarity values were calculated using the Tanimoto coefficient (Tc) ^9^. Fingerprint descriptors of different design might have been selected instead, but for our proof-of-principle investigation, the relatively simple MACCS fingerprint was readily sufficient.

### Similarity vs. distance

Pairwise similarity values were transformed into distances using the formula

*distance* = 1 – *CDF*( *similarity*)

where CDF is the cumulative distribution function for an assumed normal distribution. For each compound set, the mean and standard deviation were calculated from its pairwise similarity values. The CDF was used to emphasize compound pairs with large Tc values and de-emphasize pairs with small values compared to a linear relationship.

### Network layouts

Alternative CSN layouts were generated with in-house Java programs based upon the JUNG library ( http://jung.sourceforge.net/doc/JUNG_journal.pdf). Please also see the ‘Data availability’ section.

***Fruchterman-Reingold.*** The Fruchterman-Reingold (FR) algorithm ^10^ has so far consistently been used for NSGs ^3^ and CSNs ^5^. FR is a force-directed algorithm that brings together subsets of densely connected objects and separates different subsets from each other through repulsion (until equilibrium positions are obtained). Only similarity values reaching a pre-defined threshold are considered in FR layout construction (all other similarity values are ignored). In FR-based network views, distances between compounds have no chemical meaning.

***Kamada-Kawai.*** The Kamada-Kawai (KK) algorithm ^11^, adapted herein for CSN design, is also a force-directed layout method. However, KK uses all distances derived from similarity values as input, and optimizes (threshold-independent) edge lengths with respect to inter-compound distances. Thus, the KK approach incorporates distance relationships into network layouts. In principle, KK-based networks are completely connected. Thus, edges between distant compounds might be omitted for clarity. Although all similarity values and corresponding distance relationships are considered for network construction, for selective edge display, similarity threshold values can also be applied.

As similarity-based compound networks, KK network representations are covered by the general definition of CSNs ^4, 5^ and are in the following also referred to as KK CSNs.

## Results and discussion

### Kamada-Kawai network design

The characteristic feature of the KK approach is that it takes distances derived from all pairwise similarity values quantitatively into account during network construction. The resulting layout reflects relative compound distances, which principally increases the chemical information contained in KK CSNs compared to threshold-dependent FR CSNs. Independent of the KK network structure, which remains constant, edges in KK CSNs can be selectively displayed at varying similarity threshold values to optimize the clarity of the presentation.

### Kamada-Kawai network of a model data set

**Figure 1.**
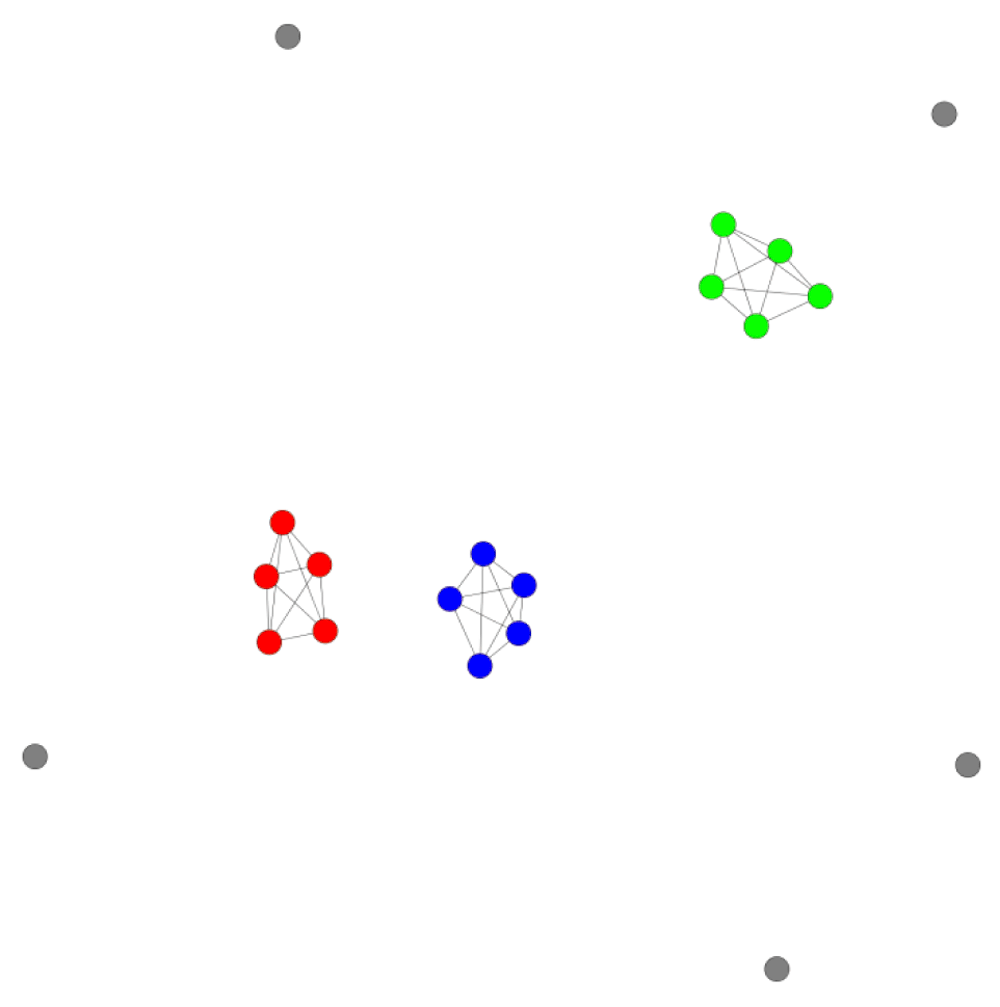
Kamada-Kawai network of a model data set. Shown is the KK CSN of the model data set according to Table 2. Data points are colored on the basis of compound subset membership: A, blue; B, red; C, green; D, gray.

For an initial proof-of-principle assessment, a model data set was generated by combining four subsets (A–D) of five hypothetical data points, each with well-defined intra-set similarity value ranges, as reported in Table 2. Subsets A–C contained highly similar data points with varying inter-subset similarity values ( Table 2), whereas subset D consisted of dissimilar data points (singletons). The KK CSN of this model data set is shown in Figure 1. All three subsets of similar data points formed separate clusters in the network, whereas data points from subset D were widely distributed. Furthermore, clusters of subsets A and B, which displayed largest inter-subset similarity values ( Table 2), were located close to each other and removed from the less similar subset C. Moreover, the KK CSN also correctly accounted for the smaller distance between A and C compared to B and C. Thus, the KK CSN incorporated for various distance relationships present in the model set; an encouraging finding.

**Table 2.** Similarity relationships in a model data set.

	A	B	C	D
**A**	1.0-0.9	0.8-0.7	0.6-0.5	0.1-0.0
B	0.8-0.7	1.0-0.9	0.4-0.3	0.1-0.0
**C**	0.6-0.5	0.4-0.3	1.0-0.9	0.1-0.0
D	0.1-0.0	0.1-0.0	0.1-0.0	0.1-0.0

For each subset of compounds in the model data set, intra-set (diagonal) and inter-set MACCS Tc value ranges are given.

### Kamada-Kawai networks for different sets of bioactive compounds

**Figure 2.**
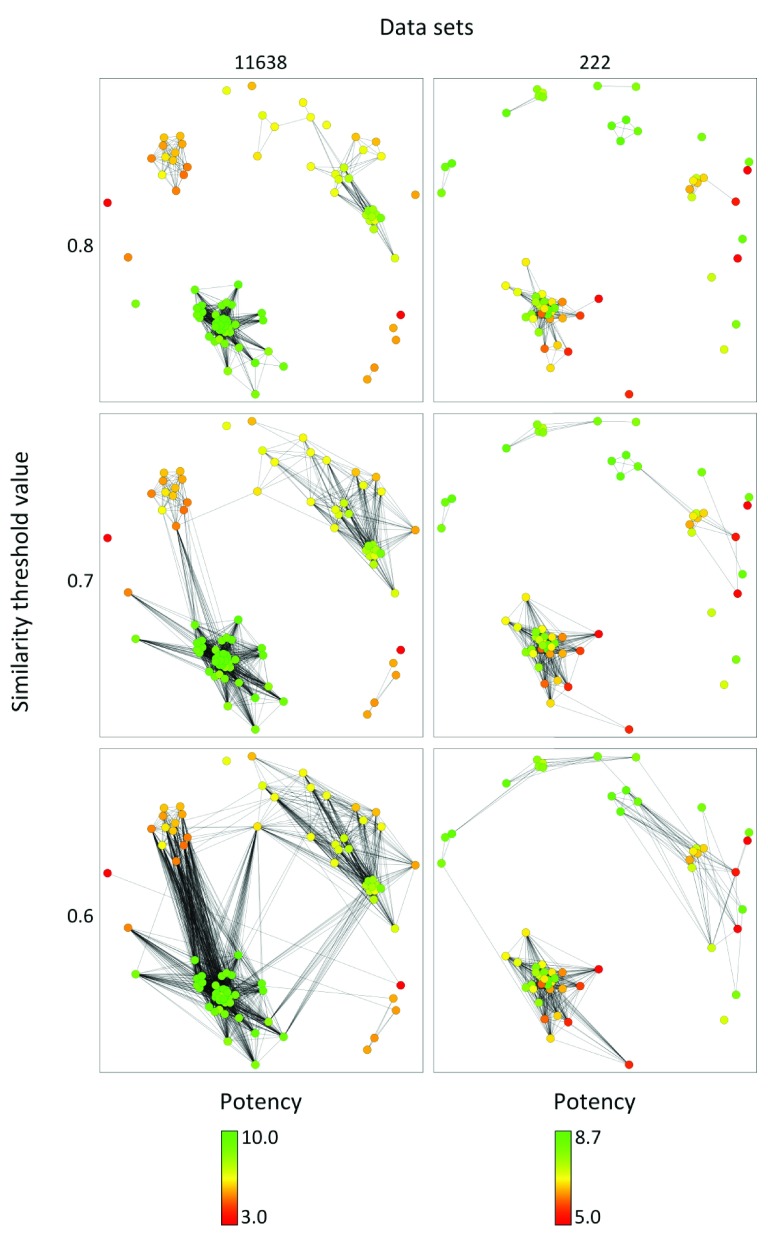
Chemical space networks with compound distance relationships. KK CSNs are displayed for two data sets (11638 and 222 according to Table 1) at three similarity threshold values of 0.8, 0.7, and 0.6, respectively. Nodes are colored on the basis of potency values applying a color gradient from green (highest potency) over yellow (intermediate) to red (lowest potency).

Figure 2 shows KK CSNs for inhibitors of MAP kinase ERK2 and glutamate [NMDA] receptor subunit ε2 (data sets 11638 and 222, respectively) ( Table 1). In each case, edges were selectively displayed at three different similarity threshold values, which enabled viewing edge distributions on a “sliding scale”. The KK CSN of the MAP kinase inhibitor set 11638 revealed a clear clustering of similar compounds with comparably high or low potency, corresponding to the presence of locally continuous SARs ^1^. By contrast, the KK CSN of the set of ligands of glutamate [NMDA] receptor subunit ε2 revealed a cluster of highly similar compounds with large potency variations, corresponding to a high degree of local SAR discontinuity ^1^. This cluster was distant from other compounds of this set, consistent with the presence of unique structural features.

Because edge densities can be selectively displayed, KK CSNs generated for larger compound data sets can be readily analyzed. In addition, for SAR analysis, one may initially select a high threshold value to primarily focus on intra-cluster relationships and then gradually decrease the threshold to concentrate more on inter-cluster connections, as illustrated in Figure 2.

### Comparison of Kawada-Kawai and Fruchterman-Reingold networks

**Figure 3.**
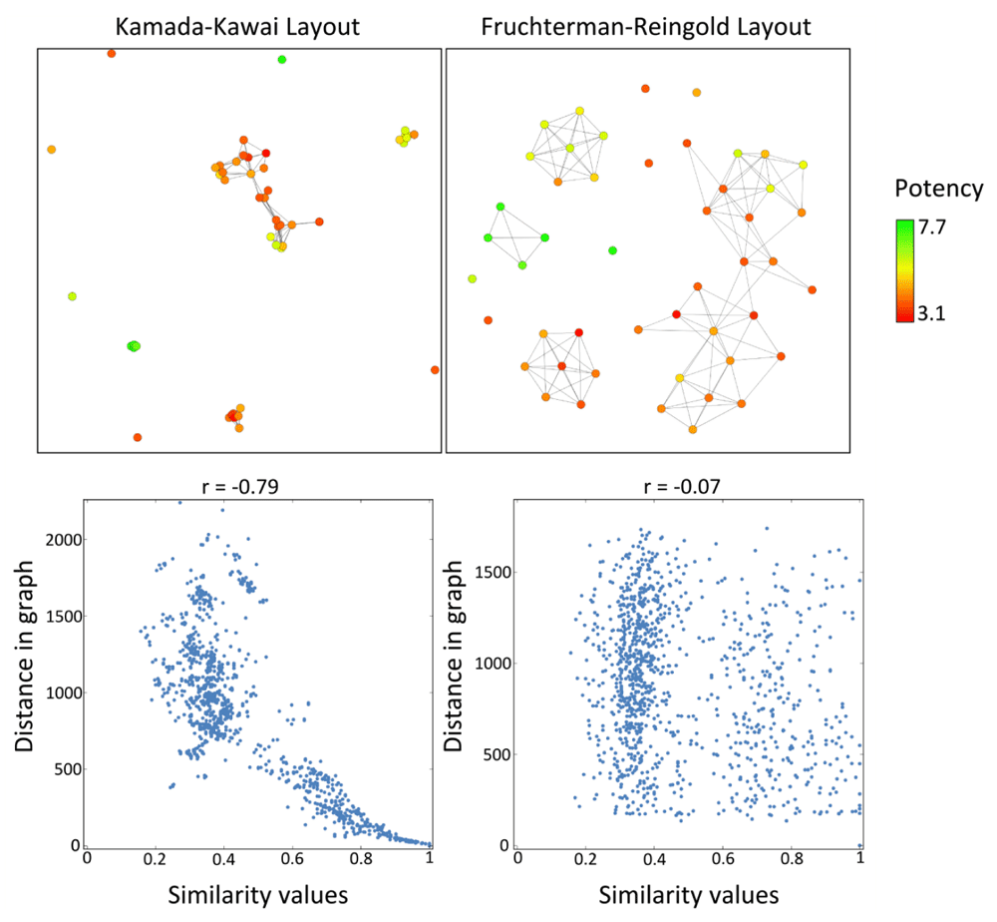
Comparison of Kamada-Kawai and Fruchterman-Reingold layouts. For data set 100476, KK and FR CSNs are compared at a similarity threshold value of 0.8 for selective edge display (KK) and network generation (FR). Nodes are colored according to Figure 2. At the bottom, similarity values and corresponding network distances of all compound pairs are compared in scatter plots and correlation coefficients are reported.

**Figure 4.**
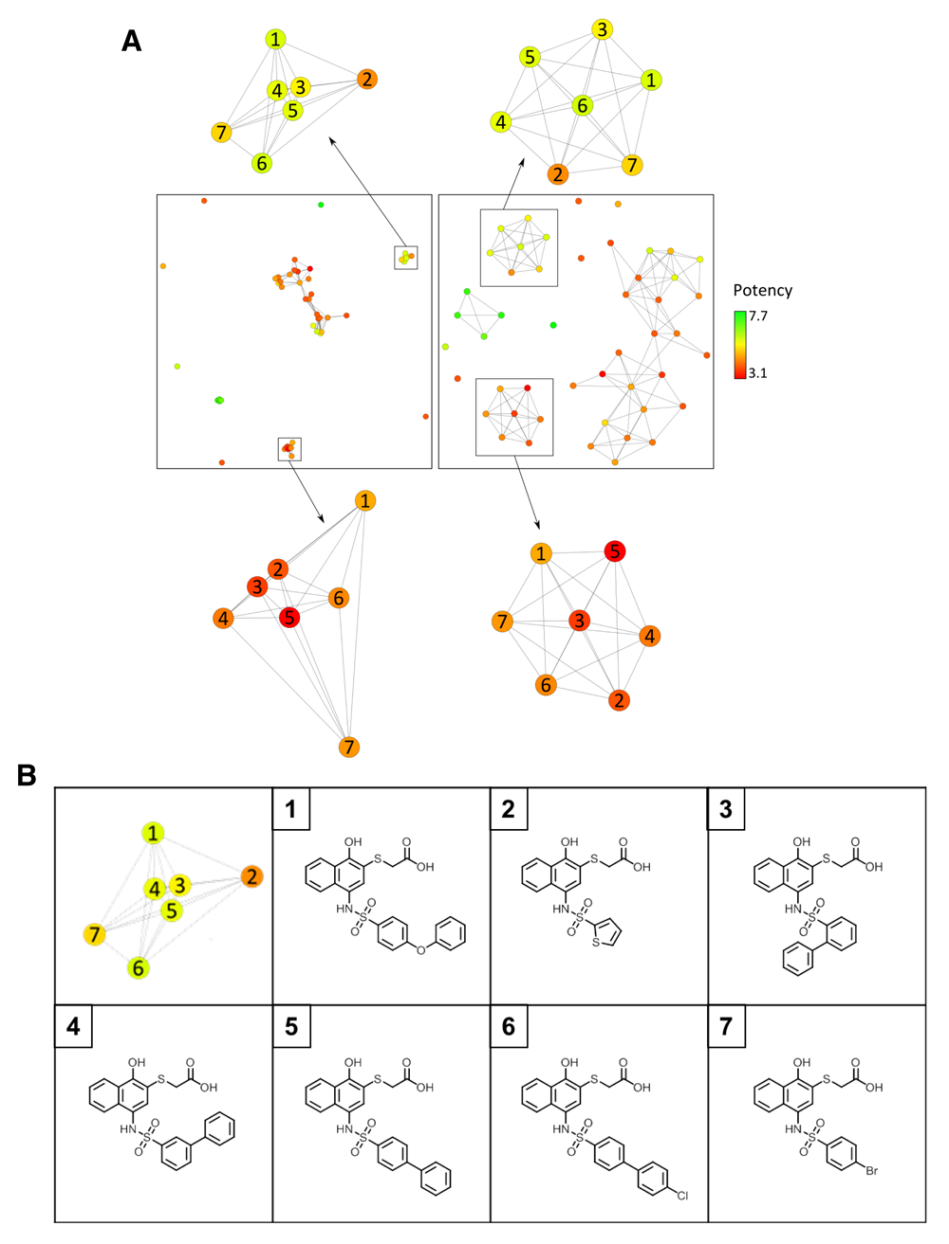
Comparison of compound communities. In ( **a**), corresponding compound communities are highlighted in the KK and FR CSNs from Figure 3 and enlarged. Compounds in each community are numbered. In ( **b**), compounds forming the top cluster in ( **a**) are shown.

Figure 3 compares the KK and FR CSNs for a set of inhibitors of the apoptosis regulator Bcl-W (data set 100476), revealing the presence of distinct layouts. In the KK CSN a larger cluster of similar –and mostly weakly potent– compounds emerged that was distant from other data set compounds. The corresponding FR CSN provided a completely different view of the compound set with several clusters that were essentially evenly distributed across the layout (consistent with its threshold-dependent force-directed design). For each of these clusters, a corresponding cluster was also identified in the KK CSN. In three cases, the corresponding compounds were so similar –and the resulting distances so small– that these clusters needed to be magnified for a detailed inspection, as shown in Figure 4a. Hence, the KK and FR CSNs also provided complementary network views of the data set.

The scatter plots in Figure 3 reveal that there was no correlation between similarity values and network distances in the FR CSN, consistent with its design principles. By contrast, with a correlation coefficient of -0.79, significant inverse correlation (i.e. large similarity values corresponding to small distances) was observed for the KK CSN, which was largely determined by compound pairs with similarity values greater than 0.5. For small similarity values, correlation was only weak. This observation was consistent with the use of the CDF in the distance function, which emphasized distance relationships between similar compounds, as discussed above. For data sets 222 and 11638 ( Figure 2), KK CSNs yielded correlation coefficients of -0.84 and -0.88, respectively.

### Comparison of compound communities and series

In Figure 4a, corresponding compound communities in KK and FR CSNs are compared in detail. FR CSN clusters contain edges of comparable length and have similar topology, which is a characteristic feature of this layout. By contrast, KK CSN clusters display different topologies and contain edges of different length that further differentiate intra-cluster similarity relationships and position similar compounds closely together. For example, compounds 3, 4, and 5 from the cluster at the top in Figure 4a only differ by the (ortho, meta, or para) position of a benzene ring and are more similar to each other than to compounds 1, 2, 6, and 7 that have different substituents ( Figure 4b).

**Figure 5.**
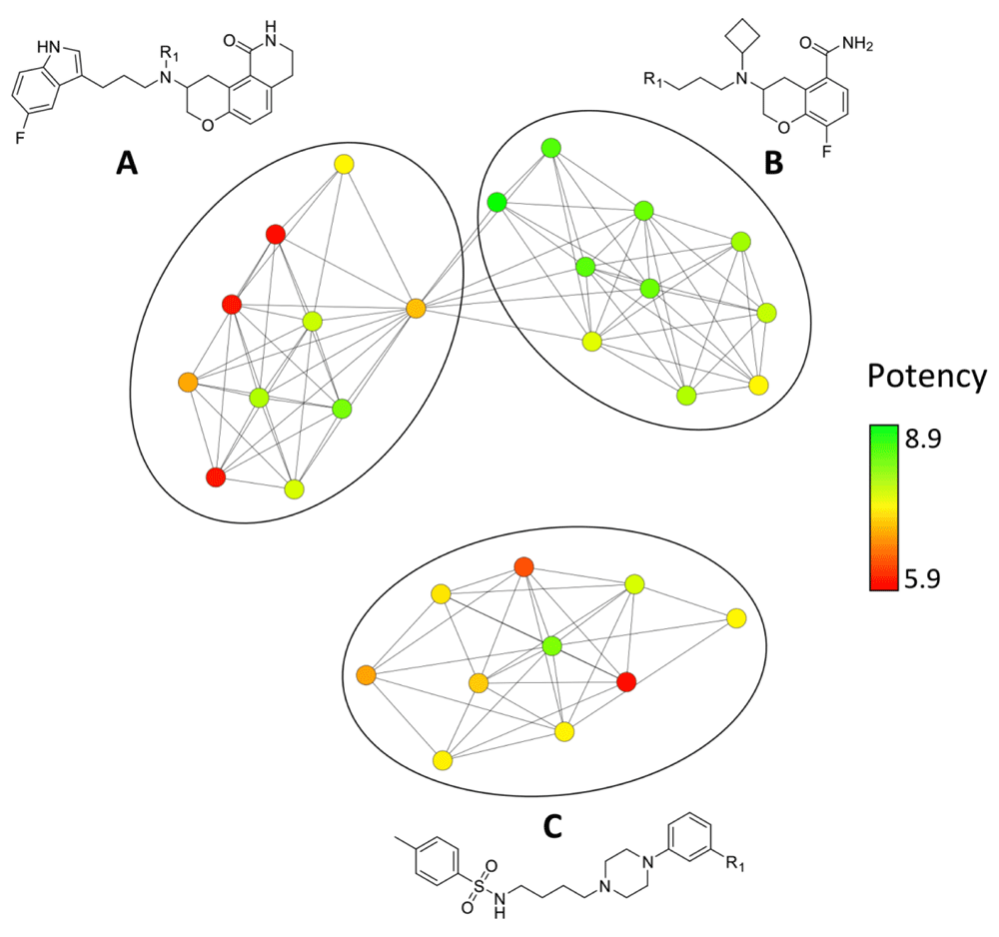
Exemplary compound series. Shown are three analog series from the KK CSN of data set 51. For clarity, a similarity threshold value of 0.88 was applied for edge display. Each analog series is encircled and its common core structure is displayed. Compounds in each series were distinguished by substituents at a single site (R1).

Figure 5 shows a KK CSN representation for three analog series (A, B, and C) that were extracted from compounds active against the serotonin 1a receptor (data set 51). Series A and B had chemically related core structures, whereas the core of series C was distinct from A and B. In the KK CSN, the three series formed communities that were separated from each other. Consistent with the structural relationship between their cores, series A and B were positioned closer to each other than to series C. A single compound from series A was found to form a bridge between the communities of A and B. This compound contained a cyclobutyl substituent at R1 and thus closely resembled the core of series B. Taken together, these observations indicated that the KK CSN captured similarity relationships between these analog series in a meaningful way.

## Conclusions

We have introduced an approach to incorporate compound distance relationships into CSNs that are coordinate-free representations of chemical space. For this purpose, the KK algorithm was adapted, which takes into account all inter-compound distances during network construction and does not depend on chosen similarity threshold values, in contrast to the FR algorithm. As such, KK networks also represent the first threshold-independent CSNs for numerical similarity measures, which further extends the current CSN spectrum. Initial results obtained for KK CSNs were encouraging, as demonstrated by the study of a model data set, for which subset relationships were correctly reproduced. Informative KK CSNs were also obtained for sets of bioactive compounds. Furthermore, we have shown that KK and FR CSNs may provide complementary representations that make it possible to view and compare compound communities in different ways. KK CSNs were also found to capture chemical relationships between analog series, which provided an advantage compared to FR CSNs.

In summary, the results of our proof-of-principle investigation suggest that KK CSNs should be of considerable interest for further exploring biologically relevant chemical space.

## Data availability

The data referenced by this article are under copyright with the following copyright statement: Copyright: © 2016 de la Vega de León A and Bajorath J

The data sets used in this study are freely available in ChEMBL ( https://www.ebi.ac.uk/chembl/) via the identifiers reported Table 1. The NSG (FR CSN) software is freely available as a part of the SARANEA program suite ^12^ in an open access deposition (DOI: 10.12688/f1000research.3713.1) ^13^. The implementation can be adapted to generate KK CSNs.
