# Supplementary material for: Design of chemical space networks incorporating compound distance relationships
Source: F1000Res. 2016 Nov 30;5:Chem Inf Sci-2634. Originally published 2016 Nov 4. [Version 2] doi: 10.12688/f1000research.10021.2 (PMC5289108; doi:10.12688/f1000research.10021.2)
Supplement: Peer review history

# Peer review: Referee response for version 2

**DOI:** 10.5256/f1000research.11126.r18086

**Published:** 2016-11-30

**Authors:** Alexandre Varnek

**Affiliations:** 1 Laboratory of Chemoinformatics, UMR 7140, CNRS (French National Center for Scientific Research), University of Strasbourg (UDS), Strasbourg, France

I have read this submission. I believe that I have an appropriate level of expertise to confirm that it is of an acceptable scientific standard.

# Peer review: Referee response for version 1

**DOI:** 10.5256/f1000research.10797.r17426

**Published:** 2016-11-22

**Authors:** Gerhard Hessler

**Affiliations:** 1 Sanofi-Aventis Deutschland GmbH., Frankfurt, Germany

Chemical space networks are an interesting method to visualize chemical space and to analyse SAR of chemical series. Recently, behavior of CSN dependent on data sets and parameter settings have been carefully analysed esp. by Prof. Bajorath and co-workers. Typically the layout of CSNs is optimized for visualization purposes and thus, distances between nodes do not have a chemical meaning. Here, a method is presented, which takes into account chemical distance information in generating the network layout, which is particularly helpful in SAR analysis. The effect of the algorithm is nicely illustrated, first with a model data set and then with real SAR data sets. In addition, a comparison between distances in the graph and similarity values shows, that the design goal to reflected compound similarity in the graph distance is achieved.

 The publication is well written, clearly structured and adds an interesting, valuable feature to CSNs.

 Some small additions might be considered by the authors. Instead of referencing the target Chembl ID in the text naming the target might make reading easier.

 Is it possible to discuss the effect of network density on layout and SAR interpretation, esp. for larger data sets?

I have read this submission. I believe that I have an appropriate level of expertise to confirm that it is of an acceptable scientific standard.

# Peer review: Referee response for version 1

**DOI:** 10.5256/f1000research.10797.r17816

**Published:** 2016-11-21

**Authors:** Alexandre Varnek

**Affiliations:** 1 Laboratory of Chemoinformatics, UMR 7140, CNRS (French National Center for Scientific Research), University of Strasbourg (UDS), Strasbourg, France

Chemical space networks (CSN) technique is an efficient way to visualize and analyze the content of chemical databases. Typically, CSNs are built using the Fruchterman-Reingold algorithm in which the distances between objects are determined for layout purposes. In this paper, for CNS construction the authors suggest to use the Kamada-Kawai algorithm providing with a graph in which the edges lengths correspond to similarity measures. Thus, CSNs obtained with the above algorithms provide with two complementary views of a chemical space. I believe that reported results are of the great interest for chemoinformatics community. The title is appropriate for the content of the article; the abstract represents a suitable summary of the work. I recommend indexing this paper as is.

I have read this submission. I believe that I have an appropriate level of expertise to confirm that it is of an acceptable scientific standard.
